# Deep molecular profiling of biliary tract cancer uncovers novel biological mechanisms and therapeutic opportunities

**DOI:** 10.1016/j.esmoop.2026.106082

**Published:** 2026-05-08

**Authors:** F. Nichetti, P. Hoffmeister, F. Korell, J. Huellein, S. Uhrig, M. Rübsam, M. Schwab, M.-V. Teleanu, M. Jenzer, S. Kreutzfeld, P. Horak, P. Konietzke, H. Glimm, C. Heining, D. Rieke, T. Kindler, C. Springfeld, C.E. Heilig, D. Hübschmann, S. Fröhling, B.C. Köhler

**Affiliations:** 1 Computational Oncology, Molecular Diagnostics Program, National Center for Tumor Diseases (NCT) and German Cancer Research Center (DKFZ), Heidelberg, Germany; 2 Department of Medical Oncology, Veneto Institute of Oncology (IOV)—IRCCS, Padua, Italy; 3 Department of Medical Oncology, National Center for Tumor Diseases (NCT) Heidelberg, University Hospital Heidelberg, Heidelberg, Germany; 4 Department of Hematology and Oncology, University Medical Center Schleswig-Holstein and University Cancer Center Schleswig-Holstein, Lübeck, Germany; 5 Department of Hematology & Oncology, University Hospital Heidelberg, Heidelberg, Germany; 6 Computational Oncology Group, Molecular Precision Oncology Program, National Center for Tumor Diseases (NCT) and German Cancer Research Center (DKFZ), Heidelberg, Germany; 7 National Center for Tumor Diseases (NCT), NCT Heidelberg, a partnership between DKFZ and Heidelberg University Hospital, Heidelberg, Germany; 8 Faculty of Biosciences at University of Heidelberg, Heidelberg, Germany; 9 Department of Translational Medical Oncology, NCT Heidelberg and German Cancer Research Center (DKFZ), Heidelberg, Germany; 10 Department of Radiology, University Hospital Heidelberg, Heidelberg, Germany; 11 Department of Translational Medical Oncology, NCT Dresden and DKFZ, Dresden, Germany; 12 Center for Personalized Oncology, University Hospital Carl Gustav Carus, Technical University Dresden, Dresden, Germany; 13 Department of Translational Medical Oncology, National Center for Tumor Diseases Dresden (NCT/UCC), a partnership between DKFZ and Faculty of Medicine and University Hospital Carl Gustav Carus, TU Dresden, Dresden, Germany; 14 Helmholtz-Zentrum Dresden—Rossendorf (HZDR), Dresden, Germany; 15 Translational Medical Oncology, Faculty of Medicine and University Hospital Carl Gustav Carus, TUD Dresden University of Technology, Dresden, Germany; 16 German Cancer Consortium (DKTK), partner site Dresden, Dresden, Germany; 17 Comprehensive Cancer Center, Charité—Universitätsmedizin Berlin, Corporate Member of Freie Universität Berlin and Humboldt-Universität zu Berlin, Berlin, Germany; 18 Department of Hematology, Oncology and Cancer Immunology, Campus Benjamin Franklin, Charité—Universitätsmedizin Berlin, Corporate Member of Freie Universität Berlin and Humboldt-Universität zu Berlin, Berlin, Germany; 19 Berlin Institute of Health (BIH) at Charité—Universitätsmedizin Berlin, Berlin, Germany; 20 UCT Mainz, Johannes Gutenberg University Mainz, Mainz, Germany; 21 Department of Hematology, Medical Oncology and Pneumology, University Medical Center, Mainz, Germany; 22 Liver Cancer Center Heidelberg, University Hospital Heidelberg, Heidelberg, Germany; 23 Innovation and Service Unit for Bioinformatics and Precision Medicine (BPM), German Cancer Research Center (DKFZ), Heidelberg, Germany; 24 German Cancer Consortium (DKTK) Heidelberg, Heidelberg, Germany; 25 Pattern Recognition and Digital Medicine Group (PRDM), Heidelberg Institute for Stem Cell Technology and Experimental Medicine, Heidelberg, Germany; 26 Division of Translational Precision Medicine, Institute of Human Genetics, Heidelberg University, Heidelberg, Germany

**Keywords:** molecular profiling, homologous recombination deficiency, molecularly guided treatment, biliary tract cancer, cholangiocarcinoma, MASTER

## Abstract

**Background:**

International guidelines recommend molecular profiling for patients with advanced biliary tract cancer (BTC) eligible for systemic treatment, but the utility of deep, multi-omic approaches remains underexplored.

**Materials and methods:**

We report molecular and clinical results of patients with BTC and enrolled in the German Cancer Consortium (DKTK) Molecularly Aided Stratification for Tumor Eradication Research (MASTER) program between February 2014 and December 2021. Tumor whole-genome/whole-exome (WGS/WES) and RNA sequencing (RNAseq) were carried out according to MASTER’s standardized workflow, and eligibility for molecularly informed therapies was discussed within MASTER’s molecular tumor board. Treatments included strategies based on a composite homologous recombination deficiency (HRD) biomarker (TOP-ART score) or RNAseq-based findings (gene fusions/overexpression).

**Results:**

Among 131 registered patients, genomic and transcriptomic profiling were successful in 115 (89%) and 89 (77%) cases, respectively. Comparative evaluation of molecular profiles highlighted ultra-rare distinctive features of BTC, including an enrichment of *PTPRM* gene fusions. MASTER-informed therapy was implemented in 23 (20.0%) patients, of whom 4 were treated according to the TOP-ART score while 12 according to RNAseq-based recommendations (gene fusions and/or overexpression), including 1 case with a *MET* fusion. Median progression-free survival (PFS) on molecularly informed therapy was 4.6 (3.7-12.7) months, with a PFS ratio (δ > 1.3)-based benefit rate of 60%.

**Conclusions:**

Multi-omic profiling of BTC within the MASTER program uncovered novel therapeutic opportunities. By the identification of ultra-rare and composite biomarkers, MASTER-guided therapies resulted in clinical benefit also in heavily pretreated patients.

## Highlights

• Deep multi-omic profiling of 115 BTCs revealed novel features and actionable targets.
• MASTER-guided therapy was delivered in 20% of the cohort with a median PFS of 4.6 months.
• Detection of rare MET fusions enabled use of crizotinib leading to a marked response in CCA.
• Positivity of TOP-ART HRD status in 44% of the cohort was linked to longer OS.
• Identification of *PTPRM* fusions in BTC related to phosphatase loss as tumor-suppressor inactivation.

## Introduction

Biliary tract cancer (BTC) comprises a heterogeneous group of rare tumors originating from the gall-bladder cancer (GBC) or the intrahepatic cholangiocarcinoma (iCCA), perihilar (pCCA), or distal (dCCA) biliary tree.^1^^,^^2^ Despite advances in BTC diagnosis and treatment, prognosis remains poor, with a 5-year overall survival (OS) <10%, largely due to advanced-stage diagnosis and limited response rates to systemic therapy.^3^, ^4^, ^5^, ^6^

Large-scale genomic profiling has revealed marked inter- and intratumoral heterogeneity in BTC, uncovering targetable alterations such as *IDH1* and *BRAF* mutations, FGFR2 and NTRK1-3 fusions, ERBB2/human epidermal growth factor receptor 2 (HER2) amplifications, or high microsatellite instability (MSI-H).^7^^,^^8^ Current international guidelines therefore recommend carrying out upfront molecular profiling by next-generation sequencing (NGS) in all patients with advanced BTC.^9^^,^^10^ However, clinically utilized NGS panels, commonly covering up to ∼500 genes, typically identify high-evidence actionable targets in <15% of patients, rising to ∼40%-50% when investigational biomarkers are included.^11^, ^12^, ^13^ Consequently, most patients continue to receive standard chemotherapy following initial chemo-immunotherapy, generally with limited clinical benefit.^9^^,^^14^^,^^15^ In this light, the value of extensive molecular characterization by multi-omic approaches to better understand underlying biological mechanisms as well as to identify new molecular targets in the clinical setting is still underexplored.^16^

In this work, we report clinical and biological characteristics of patients with advanced BTC enrolled and profiled within the German Cancer Research Center/German Cancer Consortium/National Center for Tumor Diseases (DKFZ/DKTK/NCT) Molecularly Aided Stratification for Tumor Eradication Research (MASTER) program,^13^^,^^17^ highlighting novel and ultra-rare molecular features and the outcomes of molecularly guided therapies.

## Materials and methods

### Study cohort

The MASTER program (NCT05852522) is a multicentric clinically applicable platform for the prospective, comprehensive, and multidimensional characterization of young patients with treatment-refractory tumors and patients with rare cancers at DKFZ as well as the partner sites of NCT and DKTK networks. Within this program, tumor tissue is collected and, together with matched normal control samples, analyzed via whole-genome (WGS) or whole-exome (WES) as well as transcriptome (RNAseq) sequencing using established, quality-controlled, and benchmarked pipelines.^13^ Sequencing results are then discussed by a dedicated multidisciplinary molecular tumor board (MTB), which consents evidence-based recommendations for clinical management and molecularly guided therapies. While the MTB provided treatment recommendations, the patients were eventually treated and monitored at their center of origin, with tumor assessments as per local practice.

In this work, we report on all consecutive patients with advanced BTC registered in MASTER from February 2014 to December 2021. Registration and analysis were conducted using the standardized MASTER workflow as previously published.^13^ All patients signed a written informed consent for tumor and control tissue banking, molecular analysis, and the collection of clinical data under a protocol (S-206/2011) approved by the Ethics Committee of the Medical Faculty of Heidelberg University according to the Declaration of Helsinki.

Clinical variables were manually collected by clinicians or extracted from electronic health records. Actionability evidence level for each molecular alteration and corresponding proposed treatment was faithfully reported as assigned within the MTB according to NCT/DKTK criteria (levels m1A-m4, Supplementary Table S1, available at https://doi.org/10.1016/j.esmoop.2026.106082^13^). Furthermore, each alteration was mapped to the previously defined biomarker baskets, based on the cellular pathways and processes involved and defined as previously outlined.^13^ The European Society for Medical Oncology (ESMO) Scale for Clinical Actionability of molecular Targets (ESCAT) tiers, reflecting the level of evidence available at the time of the MTB discussions (2014-2021),^10^ were a posteriori annotated for target–treatment pairs in patients who received molecularly guided treatments.

The primary objective of this analysis was to investigate the value of tumor WGS/WES and RNAseq for the identification of clinically actionable biomarkers in patients diagnosed with advanced, heavily pretreated BTC. In detail, we report genomic and transcriptomic alterations of single genes [including single nucleotide variants (SNVs), small insertions and deletions (indels), somatic copy number alterations (sCNAs), gene fusions, and gene expression] as well as composite biomarkers [including tumor mutational burden (TMB), MSI (as defined in Horak et al.^13^ and Eshleman and Markowitz^18^), and homologous recombination deficiency (HRD)] captured by tumor WGS/WES and RNAseq, and corresponding MTB recommendations for molecularly guided treatments. As secondary objectives, we report clinical patient outcomes in terms of OS of the whole study cohort, as calculated from the date of MTB discussion to death, progression-free survival (PFS), as calculated from the date of treatment start to disease progression (PD) or death, objective response rate (ORR, i.e. the proportion of patients with complete or partial response), and disease control rate (DCR, i.e. the proportion with complete response, partial response, or stable disease) by RECIST v1.1 in patients treated with molecularly informed therapies. Patients who had not undergone PD or death at the time of data cut-off were censored at their last follow-up.

### Bioinformatic analyses

Genomic and transcriptomic data were preprocessed according to the DKFZ/NCT pipeline (details concerning processing of tumor and control specimens and technical details of the WGS/WES and RNAseq analyses are described in the supplementary methods of Horak et al.^13^), and are routinely collected within a custom binary R object. From this, BTC cases were selected according to a manually curated list of patient identifiers (PatientIDs) and after an expert pathology review confirming tumor histology. Samples with insufficient tumor cell content (predefined by pathologist) or severe degradation were excluded. Moreover, only one sample (TumorID) per patient was analyzed, prioritizing higher purity and availability of RNAseq data.

Descriptive analysis of SNVs, indels, sCNA (namely gene amplifications—termed ‘gain’ in the bioinformatic pipelines—and losses) and gene fusions were analyzed*,* and only SNVs and indels that were classified according to Mayakonda et al.^19^ were retained. A BTC-specific curated list of 60 genes of interest (Supplementary Table S2, available at https://doi.org/10.1016/j.esmoop.2026.106082) was curated, with the type of event (51 SNVs/indels, 19 CNVs, and 10 fusions/rearrangements) annotated based on published evidence and MASTER/ESCAT biomarker catalogs.^20^ In particular, biomarkers identified by the ESCAT as associated with improved outcomes (tier I) or antitumor activity (tier II) for BTC,^20^ as defined in supplementary table 2 of Vogel et al.,^9^ are detailed.

Co-occurrence and mutual exclusivity of genomic alterations occurring in three or more samples were estimated using a shifted Poisson binomial model adapted from the Rediscover R package.^21^

Mutational signatures for single base substitutions (SBSs) (COSMIC, version 2) were extracted using YAPSA (v 1.30.0).^17^ HRD was evaluated according to the TOP-ART score,^22^^,^^23^ a composite biomarker predicting the functional status of the homologous recombination repair (HRR) pathway in a tumor/control sample pair. It incorporates the analysis of HRR impairments based on functional germline and somatic alterations in an extensive list of HRR-associated genes (Supplementary Table S3, available at https://doi.org/10.1016/j.esmoop.2026.106082) as well as the analysis of the genomic patterns that arise as a consequence of HRD by measuring exposure to mutational signature 3 and large-scale genomic events. The individual measures are scored and combined in a scoring schema.^24^ Samples with three or more points and detected genomic patterns are considered biomarker positive and thus HRD.

Gene fusions were identified from RNAseq data using the Arriba (version 0.8, RRID:SCR_025854) pipeline.^22^^,^^23^ Count data were used to carry out differential expression analysis with DESeq2 R (version 1.42.1). Results were further processed for gene set enrichment analysis considering the Hallmark pathway database.^25^ Transcription factor activity was inferred using the metaVIPER (version 1.36.0) algorithm as previously described.^26^ Data visualization used the ggplot2, ComplexHeatmap, and enrichplot packages in R statistical software (version 4.4.2; R Core team 2021, Vienna, Austria).

### Statistical methods

Clinical and molecular characteristics were summarized using standard descriptive statistics. Differences between different patient groups were evaluated using Fisher’s exact test. OS and PFS were analyzed by Kaplan–Meier methods with log-rank tests. Additionally, to further account for cohort heterogeneity in terms of prior treatments, an exploratory analysis, calculating OS from the time of initial diagnosis and considering the start of targeted therapy as a time-dependent covariate, was carried out. Univariable and multivariable (considering prior surgery, primary tumor location, number of prior treatment lines, and age) Cox regression analyses were carried out to account for cohort heterogeneity in terms of patient- and disease-related characteristics.

PFS ratio, defined as PFS2 (PFS on the MASTER’s guided treatment)/PFS1 (PFS on last prior treatment), was analyzed using the kernel-based Kaplan–Meier method, with a predefined efficacy threshold (δ = 1.3).^27^

Enrichment of *PTPRM* fusion was identified using Fisher’s exact test. Two-tailed *P* values <0.05 were considered significant and adjusted for multiple comparisons using the Benjamini–Hochberg procedure. All statistical analyses were carried out in R [version 4.4.2 (31 October 2024), RRID:SCR_000432, Posit open source data science company] and GraphPad Prism 8.0 (GraphPad, San Diego, CA, RRID:SCR_000306).

## Results

### The MASTER BTC cohort

**Figure 1:**
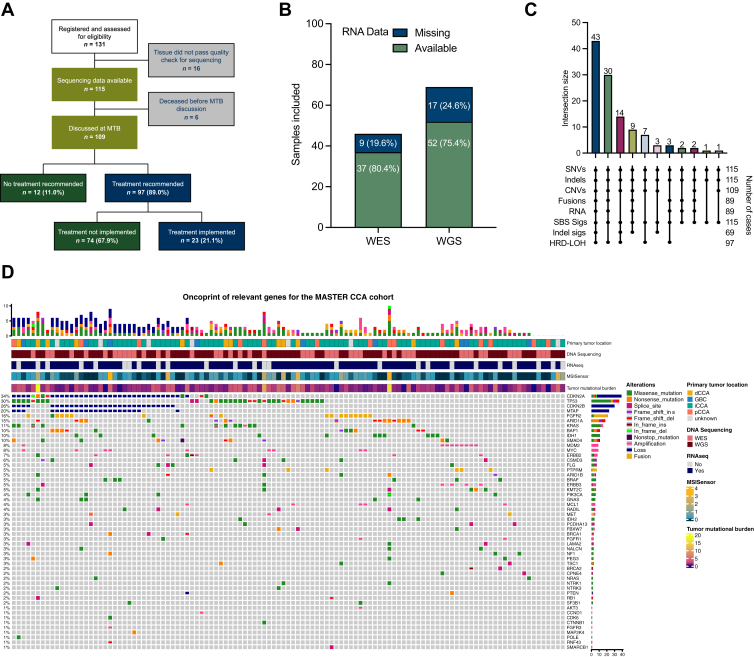
**Multi-omic analysis of biliary tract cancer (BTC) Molecularly Aided Stratification for Tumor Eradication Research (MASTER) cohort.** (A) Study flowchart depicting assessment of patients with BTCs within the MASTER cohort. (B) Availability of RNA sequencing (RNAseq) among samples profiled with whole-exome sequencing (WES) and whole-genome sequencing (WGS) data. Bars represent the number of samples for which RNA was available or missing in each group. (C) UpSet plot showing the distribution of samples with various types of genomic and transcriptomic data available. Vertical bars indicate the number of samples present in each combination of data types, while horizontal bars show the total number of samples available for each individual data type. Data types include single nucleotide variants (SNVs), insertions/deletions (indels), copy number variants (CNVs), gene fusions/rearrangements (fusions), RNAseq (RNA), single base substitution mutational signatures (SBS sigs), indel-based mutational signatures (indel sigs), and homologous recombination deficiency scores (HRD-LOH). (D) OncoPrint summarizing genomic alterations in selected BTC-relevant genes across the BTC MASTER cohort. Each column represents a patient sample, and each row corresponds to a gene. Annotations below the plot indicate clinical and molecular features including DNA sequencing method (WES or WGS), availability of RNAseq, microsatellite instability status (MSISensor score), and tumor mutational burden (TMB). Frequencies of alterations across the cohort are shown as percentages on the right. dCCA, distal cholangiocarcinoma; GBC, gall-bladder carcinoma; iCCA, intrahepatic cholangiocarcinoma; pCCA, perihilar cholangiocarcinoma.

Between February 2014 and December 2021, 131 patients with an International Classification of Diseases, 10th Revision (ICD-10)-based diagnosis of BTC were enrolled in MASTER, with a total of 141 tumor samples available for quality checks. Sixteen cases were discarded from the analysis because severe degradation or insufficient tumor cell content was observed in the tumor sample, which did not allow reliable molecular profiling, and in 10 patients two samples from different biopsy sites were profiled. The final cohort comprised 115 (87.9%) cases with successful genomic profiling, of whom 89 (67.9%) also had RNAseq data available. The Consolidated Standards of Reporting Trials (CONSORT) study flowchart is depicted in Figure 1A. In detail, WGS and WES were performed in 69 (60.0%) and 46 (40.0%) tumors, with RNAseq data available for 52 (75.4%) and 37 (80.4%) cases, respectively (Figure 1B).

Clinical patient and tumor characteristics are summarized in Table 1. The majority had iCCA (73, 63.5%) and seven had unspecified CCA (6.1%, i.e. no precise clinical annotation was available on the primary tumor location). Patients received a median of 1 (range 0-6) therapy line before MASTER registration. Of note, two cases were identified with mixed hepatocellular carcinoma (HCC)–CCA tumor—a rare type of liver malignancy sharing features of both iCCA and HCC.^28^^,^^29^ Investigated BTC cases had a median time from diagnosis to registration of 6.8 (range 0.2-70.2) months. Median time from registration to last follow-up date was 8.4 (range 0-60.2) months. Sixty-nine (53%) patients were deceased at the time of last follow-up.

**Table 1:** Characteristics of the cohort

Characteristic	*N* = 115
Sex, male, *n* (%)	69 (60)
Age at diagnosis, median (IQR), years	46 (37-52)
Primary tumor location, *n* (%)	
iCCA	73.0 (63.5)
pCCA	14.0 (12.2)
dCCA	12.0 (10.4)
GBC	9.0 (7.8)
Unknown	7.0 (6.1)
Histology, *n* (%)	
Cholangiocarcinoma	110 (95.6)
HCC–CCA	2 (1.7)
Other^a^	3 (2.6)
Prior surgery on primary tumor, *n* (%)	61 (53)
Number of prior lines of treatment, median (range)	1 (0-6)
MTB discussed, *n* (%)	109 (94.9)
Received molecularly guided treatment, *n* (%)	23 (20.0)

e/iCCA, extra/intrahepatic cholangiocarcinoma; GBC, gall-bladder carcinoma; HCC, hepatocellular carcinoma; IQR, interquartile range; MTB, molecular tumor board; p/dCCA, perihilar/distal cholangiocarcinoma.

a Other histology includes adenosquamous carcinoma and mucinous carcinoma.

### Molecular landscape and clinical outcomes

Genomic profiling resulted in the successful analysis of SNVs and indels (115 cases, 100.0%), CNVs (109, 94.8%), fusions/rearrangements (89, 77.4%), and composite biomarkers like TMB, MSI status, SBS-based mutational signatures (115, 100.0%), indel-based mutational signatures (69, 60.0%), and HRD (97, 84.3%) (Figure 1C). Variant classification as well as variant types are depicted in Supplementary Figure S1A-C, available at https://doi.org/10.1016/j.esmoop.2026.106082. A median of 63 variants per sample was observed, including 58 [interquartile range (IQR) 39–99] SNVs and 2 (IQR 1-4) indels (Supplementary Figure S1D, available at https://doi.org/10.1016/j.esmoop.2026.106082). Types of SNVs are listed in Supplementary Figure S1E, available at https://doi.org/10.1016/j.esmoop.2026.106082. The most frequently mutated genes were *TP53* (30%), *ARID1A* (15%), *IDH1/2* (10%), *BAP1* (10%), and *KRAS* (10%), as expected from previous cohorts^7^^,^^30^ (Supplementary Figure S1F, available at https://doi.org/10.1016/j.esmoop.2026.106082). A curated list of genes of interest (Supplementary Table S2, available at https://doi.org/10.1016/j.esmoop.2026.106082) was used to portray the molecular landscape of the cohort (OncoPrint in Figure 1D). Among ESCAT tier I/II biomarkers, *IDH1* mutations were identified in 11 (9.6%) patients and *FGFR2* fusions in 16 (13.9%), of whom 1 patient (0.9%) harbored a concurrent amplification; *ERBB2* (HER2) amplifications were found in 5 (4.3%) patients, while 6 (5.2%) patients had mutated *BRAF*, of whom 3 had class I V600E mutations. No *KRAS* G12C mutations were found.

Significant genomic associations included co-occurrence of FGFR2 fusions with *BAP1* mutations, MYC with MCL1 amplifications, and TP53 with *KRAS* mutations. Conversely, *TP53* mutations were mutually exclusive with *CDKN2A/B* loss. *MTAP* loss significantly co-occurred with *CDKN2A* and *B* loss, while it was mutually exclusive with *TP53* mutations, as previously reported.^31^ ESCAT tier I-II alterations were mutually exclusive, except for *BRAF* mutations and *ERBB2* amplification, as one case reported both alterations. Of note, *MET* fusions significantly co-occurred with *TSC1* and *PCDHA13* mutations (Supplementary Figure S1G, available at https://doi.org/10.1016/j.esmoop.2026.106082).

Overall, the median number of mutations per megabase was 1.302 IQR 0.798-2.130), comparable to The Cancer Genome Atlas Cholangiocarcinoma (TCGA CHOL) cohort (pairwise *t*-test, *P* = 0.995, Supplementary Figure S2A, available at https://doi.org/10.1016/j.esmoop.2026.106082). In total, three (2.6%) samples had a high TMB (>10 mutations/Mb) (histogram at the top of Figure 1D). Among composite biomarkers, two (1.7%) cases were identified as MSI-H, of whom one (0.8%) was associated with high TMB.

**Figure 2:**
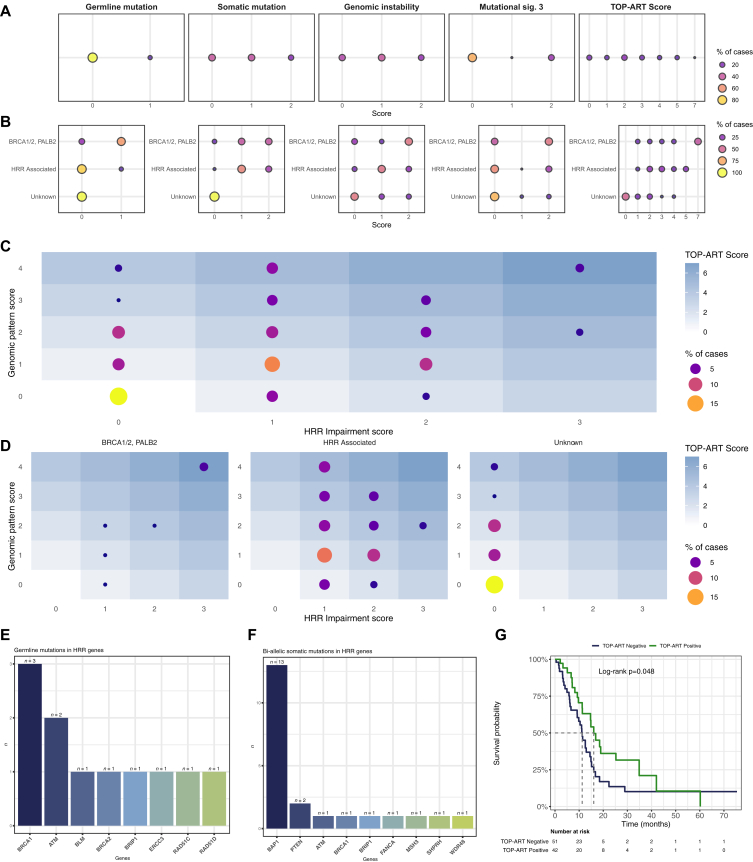
**Homologous recombination repair (HRR) and deficiency (HRD) landscape and clinical relevance in the cohort.** (A) Distribution of HRD-related features (germline and somatic mutations, genomic instability, MutSig3, and composite TOP-ART score) by score and frequency (% of cases). (B) Stratification of HRD scores based on gene category: BRCA1/2 and PALB2, HRR-associated genes, and genes of unknown association. (C) Relationship between genomic pattern score (genomic instability + MutSig3) and HRR impairment score (germline + somatic mutations), with dot size representing proportion of cases. (D) Relationship between genomic pattern score and HRR impairment score, stratified by gene category. (E) Frequency of germline mutations in HRR-related genes. (F) Frequency of bi-allelic somatic mutations in HRR genes. (G) Kaplan–Meier plot showing survival probability stratified by TOP-ART score status (positive versus negative).

Analysis of mutational signatures highlighted that mutational signature 3 (mutSig3/AC3/SBS3), a genomic signature associated with HRD, was present in 41 (35.6%) cases (Supplementary Figure S2B, available at https://doi.org/10.1016/j.esmoop.2026.106082), of which 27 (23.5%) were identified with high confidence [95% confidence interval (CI) of the predicted exposure not including zero]. The functional status of the HRR pathway could be evaluated in 97 (84.3%) cases and included germline and bi-allelic somatic mutations in HRR-related genes as the HRR impairment score, and HRD-related loss of heterozygosity, large-scale state transitions, and mutSig3/AC3 exposure as the HRD genomic pattern score (Figure 2A-D).

The resulting median TOP-ART score, as defined by Rübsam et al.,^24^ was 2. Biomarker positivity (see ‘Materials and methods’ section) was detected in 43 (44.3%) cases; a score ≥5 was identified in 15 (15.5%) patients, and 3 (3.1%) patients had a score of 7; 11 (11.3%) patients had germline mutations contributing to the score, of whom 4 (4.1%) had g*BRCA1/2* mutations (Figure 2E). Fifty-eight (59.8%) patients harbored HRR-related somatic mutations, of whom 21 (21.6%) had all gene copies (corresponding to bi-allelic alterations in germline), with *BAP1* being the most affected gene (*n* = 13) (Figure 2F). We next explored scoring differences according to the presence of germline and/or somatic alterations in the core HRR genes (*BRCA1/2, PALB2*) or other HRR-associated genes. As expected, cases with core HRR mutations had higher genomic pattern scores (i.e. genomic instability + mutSig3/AC3 exposure) and overall TOP-ART scores (Figure 2C and D). Nonetheless, strong genomic patterns were also detected in samples without core HRR-related gene mutations and even in the absence of detected causal alterations, confirming the utility of an ultra-deep molecular profiling in these tumors and the potential of composite biomarkers. Of note, a TOP-ART positivity was significantly associated with longer OS (Figure 2G).

We next examined the distribution of clinically actionable biomarkers and molecularly informed recommendations. Six cases were not discussed at the MTB, as the patients had deceased before the meeting. Of the remaining 109 MTB-discussed cases, 97 (89.0%) received at least one molecularly informed recommendation for clinical management. A median of 5 (range 1-28) unique molecular targets were identified per patient, with the most frequent being FGFR2-4 overexpression and *FGFR2* fusions, exposure to mutSig3/AC3, deletions in *CDKN2A-B, BAP1*, and *KRAS* SNVs/indels (Supplementary Tables S4 and S5, available at https://doi.org/10.1016/j.esmoop.2026.106082). The most frequent type of clinically actionable alterations was RNAseq-based gene overexpression (29.5%), followed by SNVs/indels (17.2%) (Supplementary Table S6, available at https://doi.org/10.1016/j.esmoop.2026.106082); 85.7% of all identified targets were based on somatic alterations, 10.8% from composite biomarkers, and 3.5% from germline alterations only. Details are depicted in Supplementary Figure S3A, available at https://doi.org/10.1016/j.esmoop.2026.106082. Most biomarkers fell into the treatment baskets tyrosine kinases (TKs, 34.1%), DNA damage repair (DDR, 24.8%), and cell cycle (CC, 11.1%) (Supplementary Table S7 and Figure S3B, available at https://doi.org/10.1016/j.esmoop.2026.106082).

**Figure 3:**
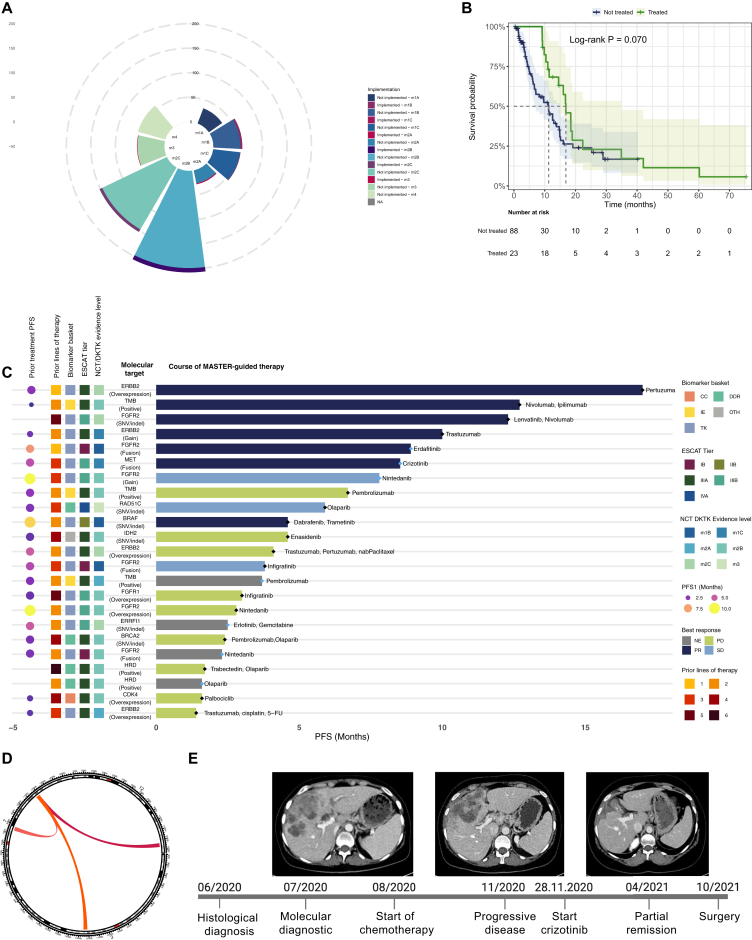
**Molecularly guided therapy following MASTER’s MTB recommendation.** (A) Proportion of recommended and implemented treatments according to NCT/DKTK evidence levels. (B) Kaplan–Meier curves for overall survival according to implementation of MASTER’s guided therapy. (C) Swimmer plot representing progression-free survival (PFS) under MASTER-guided therapy in patients with BTC. Each bar represents an individual patient’s course of therapy, annotated with the specific therapeutic agents administered. Molecular alterations are indicated on the left, along with associated biomarker baskets, NCT/DKTK evidence levels, and the number of prior lines of therapy. Bars are colored according to best response (NE, PD, PR, SD). Duration of therapy is shown in months, with blue dots indicating censoring for disease progression at the time of analysis. (D) Circos plot visualizing *MET* fusions identified in the cohort. Chromosomal locations are arranged circularly with cytoband annotations. (E) Clinical course of a patient receiving crizotinib based on MASTER MTB recommendation. 5-FU, 5-fluorouracil; CC, cell cycle; DDR, DNA damage repair; DKTK, German Cancer Consortium; ESCAT, ESMO Scale for Clinical Actionability of molecular Targets; IE, immune evasion; MASTER, Molecularly Aided Stratification for Tumor Eradication Research; MTB, molecular tumor board; NCT, National Center for Tumor Diseases; NE, not evaluable; OTH, other; PD, progressive disease; PFS, progression-free survival; PR, partial response; SD, stable disease; SNV/indel, single nucleotide variant/insertion-deletion; TMB, tumor mutational burden; TK, tyrosine kinase.

Based on these identified biomarkers, patients received a median of 2 (range 1-6) treatment recommendations, of which 73.6% and 26.4% were single-agent and combination therapies, respectively (Supplementary Figure S3C and Table S8, available at https://doi.org/10.1016/j.esmoop.2026.106082). Analogous to the biomarkers, most suggested therapies were from the TK (32.7%) and DDR (25.5%) baskets and combination treatments were most frequently combinations of the DDR and other baskets (57.4%), mainly due to 11 patients being recommended treatment with olaparib + trabectedin in the TOP-ART trial^24^ (Supplementary Figure S2D, available at https://doi.org/10.1016/j.esmoop.2026.106082). Finally, treatment recommendations were more frequently based on NCT/DKTK evidence level m2B (31.8%) and m2C (23.2%), namely on non-prospective clinical data obtained in different histopathological entities, while only 22.7% were m1A-C (i.e. based on clinical trials or case reports conducted on the same entity). The distribution of treatment recommendations according to NCT/DKTK evidence levels and MASTER’s baskets is summarized in Supplementary Table S9, available at https://doi.org/10.1016/j.esmoop.2026.106082, and Figure 3A.

Molecularly guided therapy was applied in 23 (20.0%) patients, after a median of 2 (range 1-6) prior treatment lines. Of these, 16 (69.6%) and 7 (30.4%) patients received single-agent and combination therapies, respectively. According to MASTER treatment baskets, the majority of patients (*n* = 13, 56.5%) received TK inhibitors, with an NCT/DKTK evidence level of m2B, nearly corresponding to an ESCAT tier III-A, in 47.8% (*n* = 11) of cases. Four patients were treated according to a composite biomarker of HRD (i.e. the TOP-ART score), while 12 according to RNAseq-based recommendations (gene fusions and/or overexpression).

These patients were more frequently female and more frequently had prior surgery on the primary tumor as compared with those who did not receive targeted therapies (Supplementary Table S10, available at https://doi.org/10.1016/j.esmoop.2026.106082). Median PFS on molecularly informed therapy was 4.6 (95% CI 3.7-12.7) months, while ORR and DCR were 30% and 43%, respectively (Figure 3C). Of note, median OS (mOS) was numerically longer for this subgroup of patients as compared with all others who did not receive molecularly guided treatments (mOS 16.4 versus 6.4 months, log-rank *P* = 0.070, adjusted hazard ratio 0.59, 95% CI 0.30-1.14, *P* = 0.116, Figure 3B, Supplementary Table S11, available at https://doi.org/10.1016/j.esmoop.2026.106082). When evaluating OS from BTC diagnosis, the effect of the MASTER-recommended targeted therapy becomes diluted across patients’ treatment trajectory (time-dependent HR for patients who received targeted therapies 1.37, 95% CI 0.78-2.43, *P* = 0.276).

Median PFS ratio, available for 20/23 (86.9%) cases (as PFS1 was missing for 3 cases), was 2.22 (95% CI 0.92-4.83), with an *S*_PFSratio_ (δ = 1.3) of 0.60 (95% CI 0.31-0.80, Supplementary Figure S4A-C, available at https://doi.org/10.1016/j.esmoop.2026.106082), indicating that MASTER provided a clinically and statistically significant benefit in 60% of cases.

### Rare fusions—biologically and clinically informative gene fusions in BTC

Beyond FGFR2 fusions, rare but actionable or biologically relevant fusions were detected. These included activating *MET* fusions as well as recurrent loss-of-function fusions involving receptor-type tyrosine-protein phosphatase μ (*PTPRM*). Activating *MET* fusions were identified in 3 of 115 BTC cases (2.6%), including *GOLGB1–MET, CLIP2–MET,* and *HIP1R–MET* fusions. All were predicted to generate chimeric proteins retaining an intact *MET* kinase domain, consistent with oncogenic activation (Figure 3D, Supplementary Table S12, available at https://doi.org/10.1016/j.esmoop.2026.106082). Of note, the *GOLGB1–MET* case had a mixed HCC–CCA histology; the CCA component was predominant and driving tumor aggressiveness according to pathologist review. Based on these observations, the MTB recommended treatment with *MET* inhibitors. Figure 3E summarizes the case of a patient with locally advanced, inoperable iCCA harboring a *CLIP2–MET* fusion. The patient received nine cycles of first-line chemotherapy for 3 months (from August to November 2020), showing primary resistance to chemotherapy. After enrolment in MASTER, tumor profiling detected a fusion between exon 10 of *CLIP2* (ENST00000223398.6) and exon 15 of *MET* (ENST00000318493.6), which was accompanied by *MET* amplification and overexpression, alongside a homozygous *CDKN2A/B* loss and a missense mutation in *FLG*. Subsequent treatment with crizotinib, as recommended by the DKFZ/NCT/DKTK MASTER MTB, was started in November 2020 and led to a profound (>50% reduction in longest lesion diameter) partial response after 5 months. After multidisciplinary discussion, the residual tumor was surgically removed in an individualized approach in October 2021. Taken together, these observations underpin the potential of a broad genomic characterization of BTC to identify therapeutically exploitable alterations.

In contrast to the activating nature of MET fusions, PTPRM fusions represented recurrent loss-of-function events. PTPRM fusions were significantly enriched in the BTC cohort compared with the remainder of the MASTER cohort (*P* = 0.0228, Supplementary Figure S5A, available at https://doi.org/10.1016/j.esmoop.2026.106082). PTPRM is a phosphatase involved with an underexplored but pleiotropic function.^32^^,^^33^ Within the BTC cohort, nine PTPRM fusions, mostly leading to a predicted loss of phosphatase function, were detected in seven patients (Supplementary Table S12, available at https://doi.org/10.1016/j.esmoop.2026.106082). Validating on the protein level, strongly reduced protein activity for PTPRM was inferred by applying metaVIPER algorithm in relevant cases (Supplementary Figure S5B and C, available at https://doi.org/10.1016/j.esmoop.2026.106082). Transcriptomic analysis of PTPRM fusion-positive BTCs points to pleiotropic pathway regulations including Wnt and KRAS signaling, opening a window for further functional investigation in follow-up translational studies (Supplementary Figure S5D, available at https://doi.org/10.1016/j.esmoop.2026.106082).

## Discussion

Here, we have presented the results and opportunities offered by broad molecular profiling of BTC cases within the MASTER program. The use of WGS or WES, coupled with RNAseq and germline DNA profiling, allowed for the identification of actionable alterations in a significantly greater proportion of cases than with panel sequencing. Importantly, whole transcriptome sequencing further enabled detection of gene fusions, splice variants, and expression-based therapeutic targets—such as tumor-associated antigens and immune-related markers—not captured by WES alone, thereby broadening the range of potential treatment options. These included alterations in genes involved in the CC, RAF–MEK–ERK and PI3K–AKT–mTOR pathways, and mechanisms of immune evasion.^32^^,^^33^

A striking example is the identification of *MET* fusions, an extremely rare genomic event in BTC, reported in <1% of cases in Western populations.^34^ Such alterations would likely have gone undetected without broad sequencing, thus missing an opportunity to offer a targeted therapy associated with significant clinical benefit in one patient harboring this fusion. Given the rarity of these targets, their documentation is critical to building an evidence base that supports targeted treatment in future patients.

As a drawback, the lower-than-expected frequency of some alterations, e.g. *IDH1* mutations (∼10% of cases compared with expected 13%-20%),^6^ in our cohort may be attributable to lower tumor cellularity in some samples, which is common in BTC and reduces sensitivity for detecting low-allele-frequency SNVs. Structural variants and copy number alterations were more robustly detected because RNAseq provided orthogonal validation, whereas SNVs remain more vulnerable to purity and depth limitations.

Most clinically actionable targets identified by the MTB were derived from RNAseq, particularly gene fusions and overexpression events, underscoring the importance of transcriptomic data. However, RNA-based biomarker interpretation must evolve with the therapeutic landscape.^35^ For example, although HER2-directed agents such as zanidatamab and trastuzumab deruxtecan show activity in HER2 3+ or 2+ *in situ* hybridization (ISH) -amplified BTC, they are less effective in HER2-‘low’ disease (immunohistochemistry (IHC) 1+ or 2+ without amplification),^36^ reinforcing the need for careful biomarker selection when leveraging transcriptomic data for therapeutic decision making.

Genome- and exome-wide sequencing also enabled the evaluation of composite biomarkers not reliably assessable by targeted panels, including TMB, MSI, mutational signatures, and HRD as measured by the TOP-ART score. Notably, 44% of cases exhibited a high TOP-ART score, suggesting that a substantial subset of patients may be candidates for biomarker-driven therapeutic strategies. This provides a more robust estimate of HRD prevalence in BTC than previous studies.^37^

Nevertheless, the clinical relevance of HRD in BTC, particularly with regard to platinum sensitivity, remains uncertain: the comparative efficacy of platinum therapy in HRD versus HR-proficient tumors remains to be determined. In the present study, this question could not be answered reliably due to the heterogeneous timing of tumor sampling and molecular profiling, as in a significant proportion of cases this was carried out after first-line treatment and therefore may not accurately reflect HRR status at the start of platinum therapy.

Importantly, among patients with a high TOP-ART score who were treated with a poly (ADP-ribose) polymerase inhibitor, only two lacked canonical *BRCA1/2* or *RAD51C* alterations, and both experienced only modest clinical benefit (Figure 3C). The ongoing TOP-ART trial (NCT03127215), a prospective study comparing olaparib plus trabectedin versus physician’s choice in solid tumors selected by the TOP-ART score, will provide a definitive assessment of the predictive value of this composite biomarker beyond conventional *BRCA1/2* mutation testing.

As a result of our comprehensive profiling effort, 23 patients (∼21% of all profiled cases) received a molecularly guided treatment, partially based on evidence derived from other tumor entities. Of these, approximately one-third experienced a benefit in terms of DCR and OS. Considering the setting of pretreated patients with advanced disease, a median survival >1 year following inclusion in the MASTER program for some cases receiving molecularly targeted treatments is particularly encouraging.^38^ Taken together, these data support the investigation of deep molecular profiling earlier in the disease course to maximize therapeutic benefit, enable timely treatment adaptation, and improve survival outcomes.

In addition, while many studies have focused on delineating the molecular landscape of BTC, the extended profiling in the MASTER program uncovered distinct alterations unique to this tumor type. One such example is the enrichment of PTPRM gene fusions in BTC. These events are predicted to inactivate a receptor-type tyrosine phosphatase, consistent with functional loss observed across cancer types.^39^, ^40^, ^41^, ^42^ Although PTPRM itself is not currently druggable, its recurrent inactivation and associated activation of Wnt and MYC signaling pathways suggest potential indirect therapeutic vulnerabilities that warrant further functional and preclinical investigation. Nevertheless, our findings demonstrate that broad genomic profiling enables the discovery of tumor type-specific alterations with potential relevance for future translational and therapeutic efforts.

Our study has limitations. Patients were treated and monitored at their center of origin, introducing heterogeneity in imaging intervals and outcome assessment.^27^ Moreover, given the heterogeneity of the cohort particularly with respect to timing of MASTER’s molecular profiling, we could not robustly demonstrate a survival advantage in patients receiving MASTER-recommended targeted therapy as in prior studies,^11^^,^^43^^,^^44^ as its effect likely becomes diluted across patients’ treatment trajectory.

Most importantly, our study does not provide direct evidence for the superiority of a broader sequencing approach (WGS/WES + RNAseq) over standard targeted panels, as this is not a randomized comparison of the two strategies. Furthermore, treating distinct molecular alterations with varied inhibitors (e.g. *FGFR2* fusion with infigratinib versus FGFR2 overexpression with nintedanib) introduced complexity in interpreting efficacy. Given the nature of our cohort, treatment selection was potentially driven by the temporal availability of specific agents and access to clinical trials. Consequently, the resulting sample heterogeneity precludes robust statistical conclusions for individual drug–alteration pairs.

Nonetheless, several points can be emphasized. Firstly, at the time of this study, some now-established BTC targets (e.g. BRAF) were not yet recognized. Secondly, some alterations were successfully targeted specifically because of the multimodal analytic strategy: gene fusions and gene overexpression events were confirmed through RNAseq, which only later became standard practice, still predominantly through targeted panels. Thirdly, certain alterations, such as *MET* fusions or the HRD phenotype, are not routinely assessed even nowadays and represent novel potentially actionable findings emerging from our work. Finally, the hypothesis-generating and progress-enabling aspect of ‘going a step further’ in a rapidly evolving field by integrating novel biomarker concepts should be emphasized.

In conclusion, deep and comprehensive molecular characterization of BTC not only allows for the immediate identification of therapeutically relevant vulnerabilities but also supports ongoing scientific discovery in settings closely aligned with clinical application.
