# Supplementary material for: Deep molecular profiling of biliary tract cancer uncovers novel biological mechanisms and therapeutic opportunities
Source: ESMO Open. 2026 May 8;11(5):106082. doi: 10.1016/j.esmoop.2026.106082 (PMC13187597; doi:10.1016/j.esmoop.2026.106082)
Supplement: Supplementary Figures [file mmc1.docx]

**Supplementary Figures**


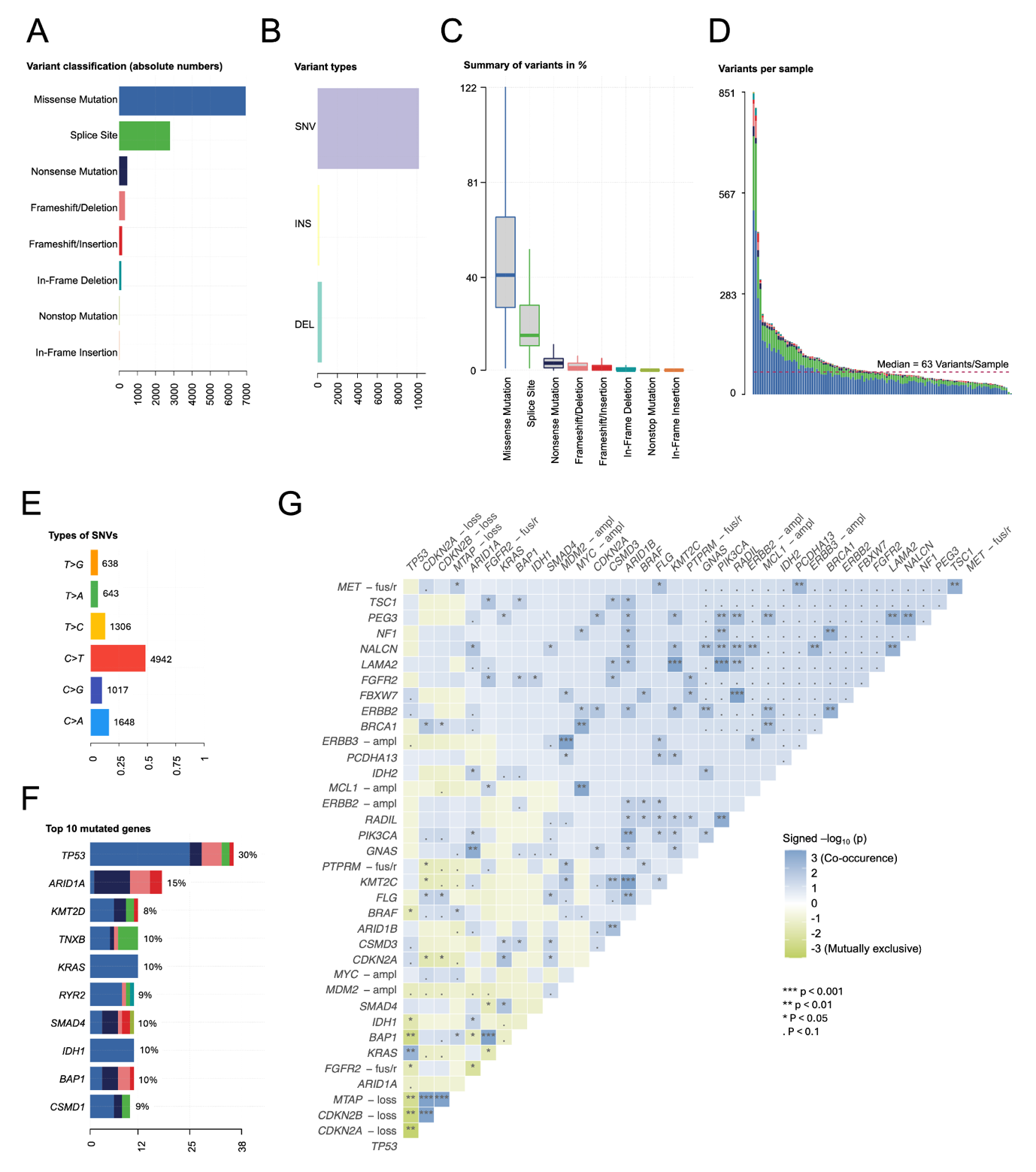


**Figure S1: Overview of molecular variants landscape in the BTC MASTER cohort. A** Distribution of variant classifications, showing absolute numbers of each mutation type, with missense mutations being most frequent. **B** Categorization of variant types: SNVs (single nucleotide variants), insertions (INS), and deletions (DEL). **C** Variants per sample, ranked by total count, with a median of 63 variants per sample. **D** Summary of variant classifications expressed as percentages across all samples. **E** Relative frequency of SNV substitution types, with C>T transitions being most common. **F** Top 10 most frequently mutated genes (SNVs / INS / DELs only) in the cohort. Color coding refers to Fig. S1A. **G** Co-occurrence and mutual exclusivity analysis of frequently altered genes. The heatmap shows pairwise relationships, where coolers indicate the type of associations (co-occurrence vs mutual exclusivity), with significance levels annotated (P < 0.1 to **P < 0.001).


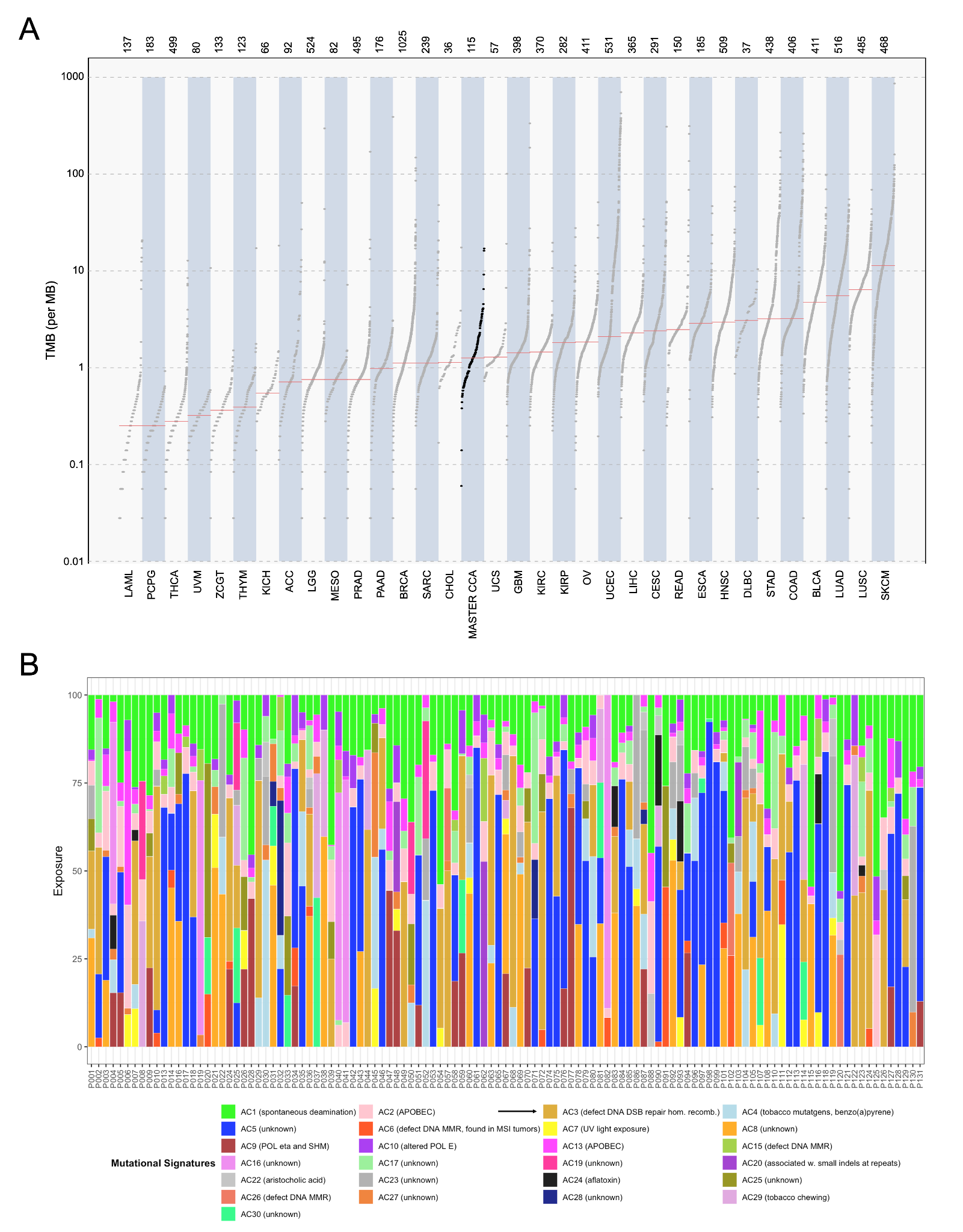


**Figure S2: Tumor mutational burden and mutational signatures across the BTC MASTER cohort. A** Tumor mutational burden (TMB) per megabase (log scale) across various TCGA tumor types and the BTC MASTER cohort (highlighted in black). Each point represents a tumor sample, with red lines indicating median TMB values per group. **B** Mutational signature contributions per sample shown as stacked bar plots. Each color represents a single-base-substitution (SBS)-based mutational signature.

**
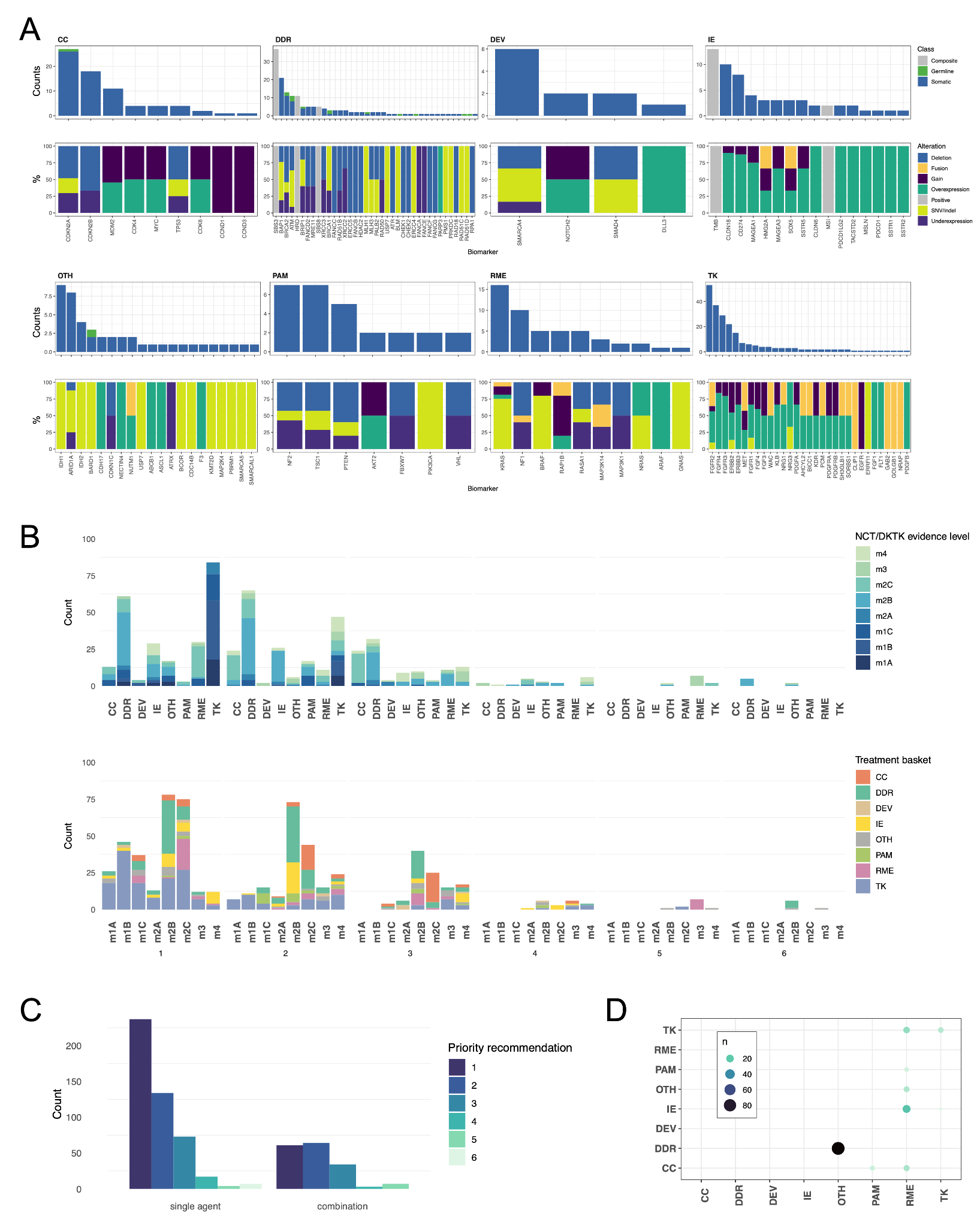
**

**Figure S3: Detailed classification of biomarkers assessed within the MASTER MTB. A** Distribution of biomarkers - colored according to the type of alteration - across treatment baskets (CC = Cell cycle, DDR = DNA damage repair, DR = developmental regulation, IE = Immune Evasion, OTH = Other, PAM = PI3K–AKT–mTOR, RME = RAF-MEK-ERK, TK = tyrosine kinases). **B** Absolute count of treatment recommendations categorized by recommendation priorities (1-6) , treatment baskets and NCT/DKTK evidence levels. **C**  Priority recommendation levels for therapies, distinguishing single-agent and combination regimens, color-coded by priority score. **D**  Dot plot summarizing the number of combination treatment recommendations between different baskets, with point size and color representing the count of cases.


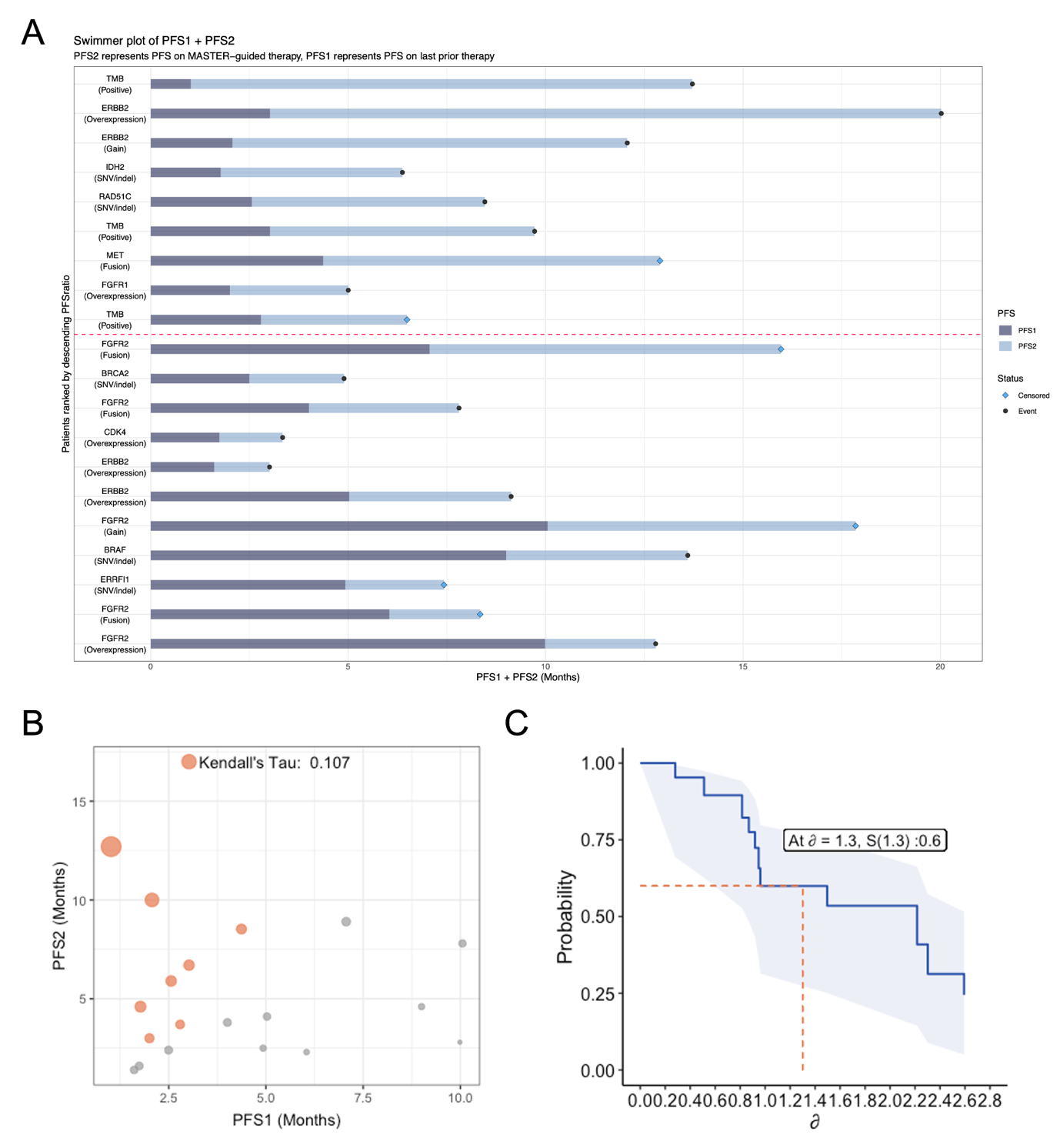


**Figure S4: PFS ratio for MASTER-recommended treatment. A** Swimmer plot of PFS1 + PFS2 ranked by descending PFSratio. PFS2 = PFS on MASTER-guided treatment; PFS1 = PFS on last prior treatment. **B** PFS2/PFS1 correlation with cases with efficient PFSratio as defined by δ=1.3 are highlighted in red. **C** Survival function estimate of PFSratio via the Kernel-based Kaplan Meier method.


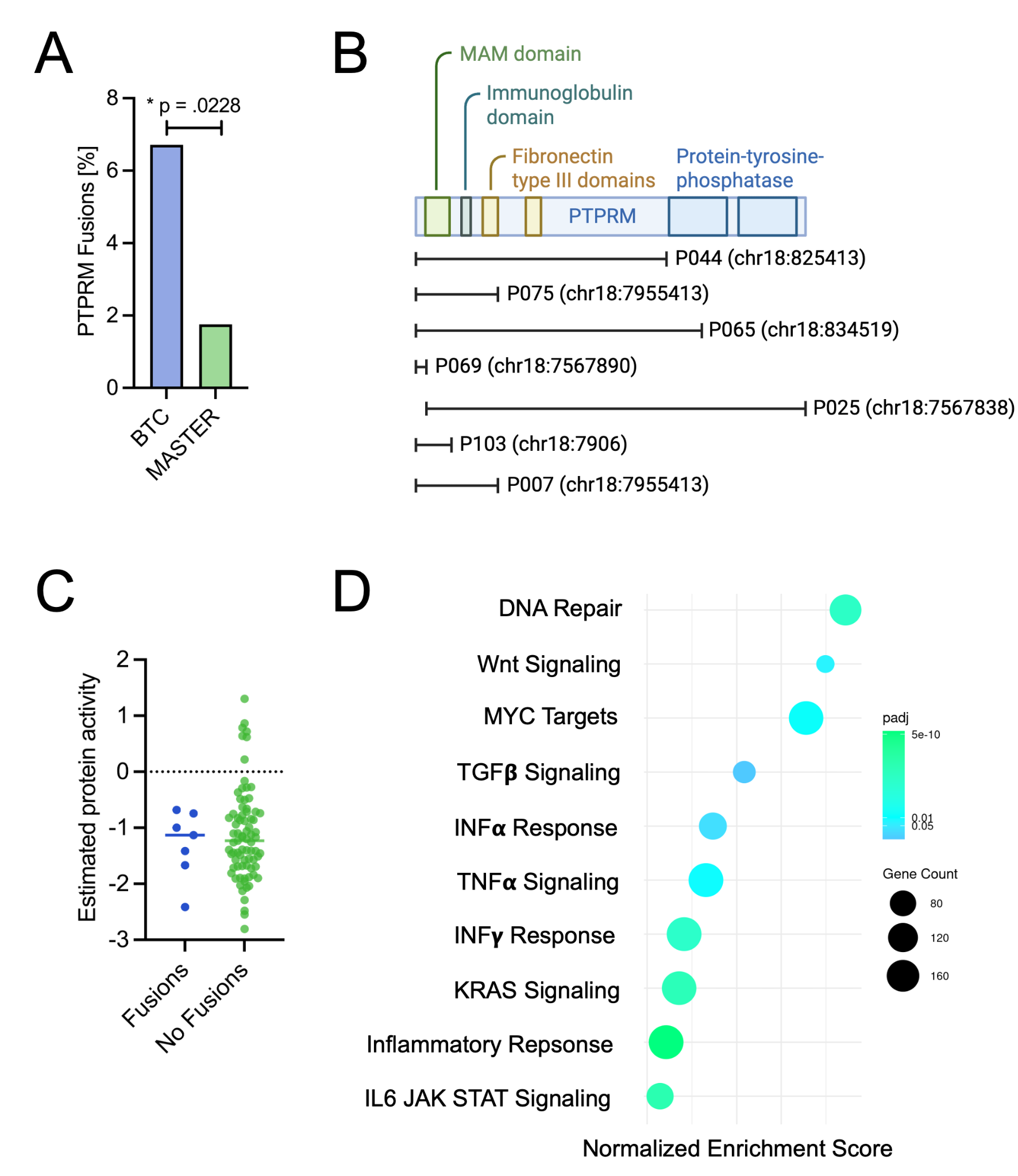


**Figure S5: PTPRM fusions in BTC. A** Cases with PTPRM fusions are significantly enriched in the BTC cohort compared to the rest of MASTER cohort (p = 0.0228, Fisher’s exact test; one fusion / patient). **B** Seven different PTPRM fusions could be observed, of which six are associated with a predicted loss of the phosphatase gene region. **C** Protein activity was estimated using mVIPER algorithm and correlated with presence of PTPRM fusions revealing far downregulated protein activity in all present PTPRM fusion cases. **D** Gene-Set enrichment analysis revealed activation of the Wnt signaling pathway and MYC related targets in the presence of a PTPRM fusion as well as downregulation of various signaling pathways such as JAK/STAT and KRAS signaling pathway.
